# The COPD-driven “lung–eye axis”: mechanisms and clinical evidence of ocular disease

**DOI:** 10.3389/fmed.2026.1893979

**Published:** 2026-08-17

**Authors:** Yuan Shen, Xin Xu, Yuxin Yang, Yuling Song, Fangwei Liu, Fangkun Zhao

**Affiliations:** 1 Department of Ophthalmology, The Fourth Affiliated Hospital of China Medical University, Shenyang, China; 2 Key Lens Research Laboratory of Liaoning Province, Shenyang, China; 3 Department of Biochemistry and Molecular Biology, China Medical University, Shenyang, China; 4 China Medical University, Shenyang, China; 5 China Medical University School of Public Health, Shenyang, China

**Keywords:** age-related macular degeneration, cataract, chronic obstructive pulmonary disease, diabetic retinopathy, dry eye, glaucoma, lung-eye axis, optical coherence tomography

## Abstract

COPD is a chronic systemic disease marked by ongoing airflow obstruction, sustained inflammation, and increased oxidative stress, whose pathological impact extends far beyond the lungs. Growing data suggest that COPD impacts the eye via chronic systemic inflammation, intermittent or persistent hypoxia, oxidative stress, and endothelial dysfunction, establishing a cross-system pathological connection termed the “lung–eye axis.” Evidence from clinical and experimental studies indicates potential associations between COPD and key ophthalmic conditions, such as dry eye disease, cataract, glaucoma, diabetic retinopathy, and age-related macular degeneration. The underlying mechanisms largely involve the retina and optic nerve, which rely heavily on oxygenation and microvascular homeostasis, highlighting them as prime ocular targets of COPD systemic effects. The application of noninvasive imaging, such as OCT and OCTA, offers quantitative and objective preliminary evidence for the lung–eye axis, indicating that ocular imaging could function as a surrogate marker of systemic disease burden in COPD. Despite being largely cross-sectional and methodologically constrained, the proposed “COPD-driven lung–eye axis” framework offers a cross-organ perspective for understanding the systemic characteristics of COPD. There is an urgent need for large, prospective, multicenter studies combining pulmonary function tests, blood gas measurements, inflammatory biomarker profiling, and multimodal ocular imaging to establish causal links and clinical utility, providing novel strategies for precision stratification and early intervention of COPD-related systemic complications.

## 1 Background

Chronic obstructive pulmonary disease (COPD) is a prevalent respiratory disease marked by persistent airflow limitation and chronic airway inflammation, with both global prevalence and mortality steadily rising (1). Conventional perspectives held that the pathological changes of COPD were largely restricted to the lungs, yet accumulating basic and clinical evidence indicates that COPD exhibits prominent systemic features, with systemic manifestations driven by chronic low-grade inflammation, increased oxidative stress, endothelial dysfunction, and chronic or intermittent hypoxia (2, 3). These pathological mechanisms are linked not only to pulmonary dysfunction but also constitute key biological foundations for the multisystem involvement of COPD, affecting organs such as the cardiovascular system, skeletal muscles, the metabolic system, and the central nervous system (2, 4).

Ocular structures, especially the retina and optic nerve, are highly metabolically active and depend on a precisely regulated microvascular circulation to sustain normal physiological function. Earlier research indicates that changes in retinal microvasculature and neural layers can sensitively mirror systemic inflammation, hypoxic stress, and endothelial dysfunction, and are thus considered an important “window” for the evaluation of systemic disorders (5, 6). On the basis of this perspective, the concept of the “lung–eye axis” was introduced to characterize the distant impact of pulmonary diseases on ocular tissues mediated by systemic inflammation, immune dysregulation, and hypoxia.

Current reviews primarily center on a broad range of respiratory diseases, while a dedicated and systematic synthesis addressing the relationship between COPD—with its distinctive features of chronic inflammation and hypoxia—and major ocular diseases remains limited. Considering the high global prevalence of COPD and its pronounced systemic pathological impact, a systematic evaluation of the evidence and potential mechanisms connecting COPD with ocular diseases from a disease-specific perspective is essential for advancing disease insight and improving clinical management. Accordingly, this review focuses on COPD, highlighting advances in research regarding its associations with major ocular diseases, examining the potential mechanisms through which COPD drives the “lung–eye axis,” and discussing its clinical relevance in order to offer new strategies for the early detection and intervention of extrapulmonary complications of COPD.

## 2 Systemic pathophysiology of COPD and ocular involvement

A substantial body of evidence indicates that the pathological impact of COPD extends beyond the airways and pulmonary parenchyma, and that it is essentially a chronic disorder with significant systemic manifestations affecting distant organs such as the cardiovascular system, skeletal muscle, metabolic system, and the central nervous system (7, 8). The systemic nature of COPD provides a critical premise for the potential linkage between COPD and changes in ocular structure and function.

**Figure 1:**
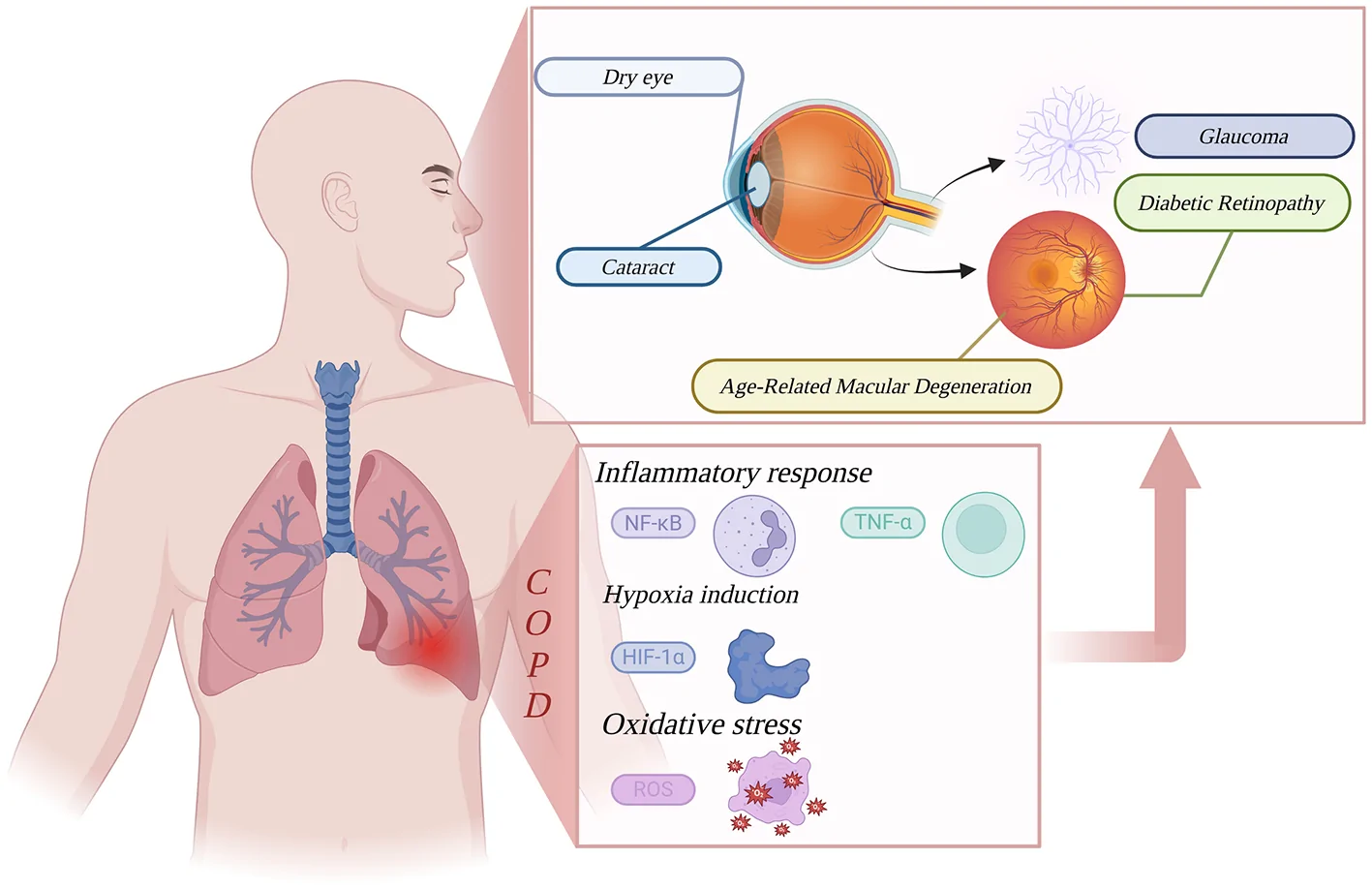
Chronic obstructive pulmonary disease impact on ocular diseases.

Current evidence suggests that chronic systemic inflammation, sustained or intermittent hypoxia, increased oxidative stress, and vascular endothelial dysfunction represent the principal pathophysiological mechanisms responsible for the multisystem manifestations of COPD (4, 9). Through the release of circulating inflammatory factors, activation of hypoxia-associated signaling pathways, and disturbances in microvascular regulation, these mechanisms can affect ocular tissues that are highly dependent on stable oxygen delivery and microvascular perfusion, thereby establishing an important pathological foundation for ocular disorders including retinal microvascular alterations and neural tissue injury (Figure 1).

## 3 Relationship between COPD and major ocular diseases

### 3.1 Dry eye disease and COPD

Dry eye disease is a multifactorial condition characterized by decreased tear film stability and inflammatory responses of the ocular surface, with its pathogenesis being closely linked to systemic inflammatory conditions. Findings from recent research suggest that dry eye disease is not solely a localized ocular surface disorder, but may serve as an “ocular surface phenotype” indicative of systemic immune and inflammatory dysregulation (10).

Individuals with COPD are chronically exposed to a low-grade systemic inflammatory milieu characterized by sustained elevations of circulating cytokines such as IL-6 and TNF-α (11), and previous studies have demonstrated that these inflammatory mediators may participate in the pathogenesis of dry eye disease by affecting lacrimal gland secretion, modifying tear film composition, and disturbing immune homeostasis of the ocular surface (12). Furthermore, chronic inflammatory processes may induce activation of apoptosis-related signaling pathways, leading to apoptosis of ocular surface epithelial cells and a reduction in goblet cells, which in turn exacerbates tear film instability and symptoms of ocular surface dryness (13).

Apart from inflammation, oxidative stress is likewise closely related to the development and progression of dry eye disease. Under physiological conditions, the ocular surface depends on antioxidant defense mechanisms to maintain redox homeostasis, whereas excessive production of reactive oxygen species (ROS) or reduced antioxidant capacity can lead to oxidative stress that damages corneal and conjunctival epithelial cells and initiates inflammatory cascades, further exacerbating tear film instability. In individuals with COPD, prolonged exposure to smoking, persistent inflammation, and mitochondrial dysfunction markedly elevate the overall oxidative burden of the body, and this systemic oxidative stress may influence the ocular surface microenvironment via the circulation, enhancing the vulnerability of corneal and conjunctival epithelial cells to oxidative injury and thus facilitating the occurrence of dry eye disease (14).

It is important to note that high-quality epidemiological studies using COPD as the principal exposure variable to systematically assess the risk of ocular surface disorders, including dry eye disease, remain limited. Current epidemiological evidence on dry eye disease is largely derived from studies of the general population or individuals with other chronic conditions, and thus cannot be directly generalized to the COPD population. While these findings do not definitively establish a causal relationship between COPD and dry eye disease, they offer important preliminary insights for further exploration of their potential pathological linkage.

### 3.2 Cataract and COPD

Cataract represents the most prevalent reversible cause of blindness globally, characterized pathologically by denaturation and aggregation of lens proteins and decreased lens transparency, ultimately resulting in optical media opacity and visual impairment. Although advancing age remains the most significant risk factor, accumulating evidence suggests that oxidative stress, chronic inflammation, and metabolic dysregulation play crucial roles in the development of lens opacity (15, 16). Against this background, the sustained oxidative stress, systemic inflammatory state, and prolonged pharmacological exposure associated with COPD may render it an important systemic factor affecting the risk of cataract formation (11).

Oxidative stress is widely regarded as a key mechanism underlying cataract formation. The lens is continuously exposed to light and a relatively high-oxygen environment, and its antioxidant defense systems, including glutathione, play a crucial role in preserving lens transparency. When the generation of ROS surpasses antioxidant defense capacity, excessive ROS can trigger oxidative modification and cross-linking of lens proteins, causing structural abnormalities and protein insolubilization, which ultimately increase light scattering and lead to lens opacification (16, 17). Individuals with COPD experience sustained oxidative stress as a result of prolonged smoking exposure, chronic activation of inflammatory cells, and mitochondrial dysfunction (11). Evidence indicates that COPD patients demonstrate decreased peripheral antioxidant capacity and increased levels of lipid peroxidation products (18). This systemic oxidative load may affect the lens microenvironment via circulatory pathways, thereby promoting the process of lens protein denaturation. Epidemiological evidence further indicates that smoking, the primary risk factor for COPD, is significantly associated with the risk of nuclear cataract, implying that oxidative injury may constitute a common pathogenic mechanism linking the two conditions (19).

Systemic inflammation may further amplify the relationship between cataract and COPD. Individuals with COPD exhibit persistently elevated levels of inflammatory mediators, including IL-6, TNF-α, and C-reactive protein in the circulation (11), which may cross the blood–aqueous barrier and affect the intraocular microenvironment, triggering local inflammation and consequently disturbing the homeostasis of lens epithelial cells. Experimental animal studies indicate that activation of inflammatory signaling pathways may induce apoptosis of lens epithelial cells and promote abnormal differentiation of lens fiber cells, thereby establishing a pathological foundation for cataract formation (20). Furthermore, the chronic hypoxia frequently associated with COPD may indirectly exacerbate lens injury, since hypoxic conditions can disrupt intracellular redox homeostasis and impair lens energy metabolism as well as antioxidant defense mechanisms, markedly increasing the vulnerability of lens tissue to oxidative damage (21).

Notably, therapeutic exposures associated with COPD management may also constitute an important risk factor for cataract formation. Prolonged or recurrent administration of systemic glucocorticoids has been shown to be significantly associated with the development of posterior subcapsular cataracts (19), and certain patients with severe COPD may require short-term or long-term systemic corticosteroid therapy during episodes of acute exacerbation. Although inhaled corticosteroids (ICS) generally exhibit lower systemic adverse effects, high-dose or prolonged use of ICS has been reported to modestly increase the risk of cataract development (22). Accordingly, assessments of the relationship between COPD and cataract should take into account both the systemic pathophysiological mechanisms of the disease and the combined influence of treatment-related exposures.

From an epidemiological standpoint, several large population-based studies have indicated that individuals with chronic pulmonary diseases exhibit a higher risk of cataract than the general population, and this association remains significant even after controlling for confounders such as age and smoking (23).

This observation suggests that COPD may participate in lens opacification through mechanisms that are independent of smoking exposure. In summary, increased oxidative stress, persistent inflammatory burden, and potential corticosteroid exposure associated with COPD may jointly affect the lens, thereby accelerating protein denaturation and loss of transparency. Although direct mechanistic evidence remains limited, cataract may be interpreted, from a systemic–ocular interaction perspective, as another potential target-organ manifestation within the “lung–eye axis” framework.

### 3.3 Glaucoma and COPD

Glaucoma represents a major cause of irreversible blindness globally, characterized pathologically by the progressive loss of retinal ganglion cells (RGCs) and thinning of the retinal nerve fiber layer (RNFL) composed of their axons, eventually resulting in optic nerve injury and permanent visual field loss (24). While increased intraocular pressure (IOP) has long been regarded as the principal risk factor, accumulating evidence demonstrates that lowering IOP alone cannot fully prevent disease progression, especially in normal-tension glaucoma (NTG), highlighting the important roles of optic nerve perfusion abnormalities, vascular autoregulatory dysfunction, and disturbances in neural metabolism in the pathogenic process (25). Consequently, glaucoma is increasingly recognized as a neurodegenerative disorder closely linked to systemic vascular conditions and oxygen metabolic regulation (26).

COPD is a chronic condition with marked systemic manifestations, whose characteristic pathological features include sustained hypoxia, systemic inflammation, and increased oxidative stress (27). Chronic inflammatory processes and oxidative stress may disrupt endothelial function and impair the autoregulatory capacity of the microcirculation, and the retinal microvasculature is regarded as a “window” reflecting systemic microvascular status, with its structural and functional abnormalities being linked to numerous systemic conditions such as cardiovascular and neurodegenerative diseases (5, 28). Under this background, COPD-related systemic vascular dysfunction may lead to reduced ocular perfusion pressure, increased vascular resistance, and decreased stability of blood flow, thereby maintaining the optic nerve head in a relatively ischemic state and increasing the susceptibility of retinal ganglion cells (RGCs) to injury.

Chronic hypoxia is considered a key pathological pathway connecting COPD with glaucoma. Increased expression of hypoxia-inducible factor-1α (HIF-1α) in the pulmonary tissue of patients with COPD suggests that the organism is exposed to chronic hypoxic stress (29). The retina represents one of the most metabolically active tissues with the highest oxygen consumption in the body, and its structural and functional integrity is highly sensitive to fluctuations in oxygen availability (30). The development of normal-tension glaucoma (NTG) is believed to be primarily associated with reduced optic nerve perfusion and dysfunction of vascular autoregulation rather than simple elevation of intraocular pressure, and this ischemia–oxidative stress network may lead to optic nerve injury even when IOP is not elevated, which is highly consistent with the pathogenic mechanisms of NTG (25, 31).

Furthermore, imaging-based studies have provided direct evidence supporting the association between these two conditions. Several investigations using optical coherence tomography (OCT) and optical coherence tomography angiography (OCTA) have demonstrated structural changes in patients with COPD, such as thinning of the retinal nerve fiber layer (RNFL), decreased peripapillary choroidal thickness, and reduced microvascular density (32–34). Earlier research indicates that thinning of the RNFL may better reflect early structural damage in glaucoma than elevated intraocular pressure alone, suggesting that neurovascular structural abnormalities associated with COPD may constitute a morphological basis for glaucoma development (35).

Epidemiological evidence has further reinforced the relationship between these two conditions. A cross-sectional analysis using Korean national health survey data demonstrated that women with restricted lung function had a significantly higher prevalence of open-angle glaucoma compared with those with normal pulmonary function, and declining lung function was positively associated with glaucoma risk (36). In addition, glaucoma patients with coexisting COPD showed lower IOP levels compared with those with glaucoma alone, indicating that impaired pulmonary function may act as a risk factor independent of intraocular pressure. In addition, other hypoxia-related conditions, including obstructive sleep apnea, have also been linked to retinal ganglion cell (RGC) damage and an elevated risk of glaucoma, further confirming the role of the hypoxia–inflammation axis in optic nerve neurodegenerative changes (37).

Overall, COPD may compromise the ability of the optic nerve to withstand perfusion variability and pressure-related stress through the synergistic effects of chronic hypoxia, systemic inflammation, and oxidative stress, thus providing a mechanistic foundation for the development of glaucoma, especially NTG. Although existing evidence is largely based on cross-sectional studies and causal relationships have not yet been established, the “lung–eye axis” framework offers a novel perspective for investigating the link between chronic pulmonary diseases and glaucoma.

### 3.4 Diabetic retinopathy and COPD

Diabetic retinopathy (DR) represents the primary cause of vision impairment in the working-age population, characterized pathologically by capillary endothelial dysfunction, breakdown of the blood–retinal barrier, microvascular occlusion, and ischemia-induced neovascularization. Recent evidence indicates that DR is not simply a microvascular complication of diabetes but a neurovascular degenerative disorder driven by chronic low-grade inflammation and oxidative stress, and its pathogenesis is influenced not only by local ocular factors but also by systemic conditions including inflammatory status and vascular health (6, 38). Within this research framework, COPD—characterized by systemic inflammation, increased oxidative stress, and chronic hypoxia—exhibits considerable pathophysiological overlap with the mechanisms involved in DR development, thereby offering a reasonable basis for a potential association between COPD and DR (4, 11).

Systemic inflammation is regarded as a key biological pathway connecting COPD with DR. In COPD patients, levels of circulating inflammatory mediators including IL-6, TNF-α, and C-reactive protein are persistently elevated, and these factors may act on retinal microvascular endothelial cells through the bloodstream, facilitating leukocyte adhesion and endothelial apoptosis to accelerate capillary non-perfusion, while inflammatory signaling simultaneously activates the nuclear factor-κB (NF-κB) pathway, initiating local inflammatory cascades that further compromise the integrity of the blood–retinal barrier (11, 39). Existing studies have confirmed that inflammatory mediator levels in the blood and vitreous fluid of DR patients are markedly increased and show a positive association with the severity of retinal lesions (39). Consequently, in individuals with diabetes complicated by COPD, the inflammatory burden imposed by COPD may synergize with the underlying inflammatory milieu of diabetes, thereby promoting the progression of retinal microvascular injury.

Oxidative stress is recognized as a core mechanism underlying the development of both DR and COPD. A hyperglycemic state can independently promote the production of ROS through mechanisms including activation of the polyol pathway, aberrant activation of protein kinase C, and accumulation of advanced glycation end products (AGEs), whereas COPD patients are chronically exposed to heightened oxidative stress due to persistent inflammation and smoking exposure, resulting in substantially increased ROS generation (11, 40). Such combined oxidative stress may amplify mitochondrial dysfunction and DNA damage, consequently heightening the sensitivity of retinal ganglion cells and capillary endothelial cells to metabolic stress. Experimental studies using animal models have confirmed that sustained oxidative stress can lead to capillary pericyte loss, a hallmark structural change in the early stage of DR (40, 41). Consequently, oxidative stress associated with COPD may further lower the resilience of retinal tissues to hyperglycemia-induced damage in diabetic patients, thereby promoting the progression of DR.

In this pathological interplay, chronic hypoxia may further exacerbate ischemia-related damage by disrupting the stability of retinal microcirculation. Retinal ischemia represents a central driver of proliferative DR, as hypoxic conditions activate hypoxia-inducible factor-1α (HIF-1α), leading to increased expression of vascular endothelial growth factor (VEGF) and consequently promoting abnormal neovascular formation (42). Persistent hypoxia and vascular endothelial dysfunction in COPD patients may markedly compromise the autoregulatory capacity of retinal microcirculation, thereby increasing the likelihood of retinal ischemia occurrence and persistence. Imaging studies using OCTA have demonstrated that COPD patients show microvascular alterations including decreased retinal capillary density and reduced perfusion (43), changes that are also observed in the early phases of DR. Such instability in retinal microcirculation may facilitate the progression of diabetic patients toward more severe stages of DR.

From an epidemiological perspective, population studies indicate that individuals with diabetes who also have chronic pulmonary disease exhibit a markedly higher risk of microvascular complications compared with diabetic patients without pulmonary comorbidities (44). Although large prospective studies specifically examining the association between COPD and DR are still scarce, multiple systemic inflammatory disorders have been reported to increase the risk of DR, indicating that systemic inflammation may play an amplifying role in retinal microvascular damage. Integrating existing mechanistic insights and clinical evidence, COPD may promote the development and progression of diabetic retinal pathology by augmenting systemic inflammatory burden, intensifying oxidative stress, and compromising the stability of microvascular perfusion. Large prospective longitudinal cohort studies and stratified analyses are needed in the future to determine the precise role of COPD across different stages of DR and to better define its clinical management implications.

### 3.5 Age-related macular degeneration and COPD

Age-related macular degeneration (AMD) represents one of the principal causes of central vision impairment among older adults, characterized pathologically by retinal pigment epithelium (RPE) dysfunction, structural alterations of Bruch's membrane, progressive photoreceptor degeneration, and abnormal choroidal vascular proliferation or atrophy. Recent evidence suggests that AMD is a degenerative disorder driven by oxidative stress, chronic low-grade inflammation, complement system abnormalities, and vascular dysregulation, and its pathogenesis is influenced not only by local ocular factors but also by systemic inflammatory status and vascular health (45, 46). The intrinsic pathological features of COPD—such as chronic hypoxia, systemic inflammation, and increased oxidative stress—offer a strong biological foundation for a potential link between COPD and AMD (11, 47, 48).

Oxidative stress is considered a key mechanism in the development of AMD. Due to its high metabolic activity and persistent light exposure, the macular region is chronically exposed to ROS, rendering retinal pigment epithelium (RPE) cells and photoreceptors particularly vulnerable to oxidative injury (45). In COPD patients, excessive ROS are persistently generated as a result of smoking exposure, inflammatory cell activation, and mitochondrial dysfunction, accompanied by a significant reduction in the efficiency of endogenous antioxidant defense mechanisms (11, 27). Such systemic oxidative stress may affect the retinal microenvironment via the bloodstream, facilitating the accumulation of lipid peroxidation products in retinal pigment epithelium cells, initiating inflammatory cascade reactions, and consequently accelerating RPE cell senescence and apoptosis (27, 45). Earlier studies have confirmed that oxidative damage stimulates RPE cells to release inflammatory mediators and vascular endothelial growth factor (VEGF), facilitating geographic atrophy progression in dry AMD and participating in choroidal neovascularization in wet AMD (45, 50).

Chronic hypoxia is another key mechanistic pathway connecting COPD with AMD. Decreased choroidal perfusion is regarded as an early pathogenic event in the development of AMD (46). Persistent hypoxia and endothelial dysfunction associated with COPD can decrease choroidal perfusion efficiency and disrupt microvascular autoregulatory capacity, rendering the RPE–photoreceptor complex more vulnerable to ischemic damage (47). Furthermore, hypoxic conditions can increase VEGF expression via activation of the hypoxia-inducible factor signaling pathway, and excessive VEGF is a principal driver of choroidal neovascularization in neovascular (wet) AMD, making this mechanism highly consistent with the pathological basis of wet AMD (50). Imaging investigations using OCT and OCTA have demonstrated that AMD patients frequently exhibit decreased choroidal thickness and capillary perfusion abnormalities (51), and comparable microvascular structural and functional alterations have also been reported in COPD populations (48), indicating that the two diseases may share similar vascular pathological features.

Epidemiological studies provide additional evidence supporting the relationship between AMD and COPD. Large population-based studies have identified smoking as one of the most significant environmental risk factors for AMD, and as the principal causative factor for COPD, creating considerable overlap in the risk factor spectrum of these two conditions (52). Furthermore, analyses of population-based databases indicate that patients with chronic pulmonary diseases have a higher risk of AMD compared with the general population, and this association remains significant even after controlling for smoking status (49). This evidence indicates that, in addition to smoking, chronic inflammation and oxidative stress associated with COPD may independently participate in the pathogenesis of AMD. Although existing evidence linking the two diseases largely originates from observational studies, the causal direction of this association has yet to be clearly established. From a mechanistic integration standpoint, COPD may create favorable pathological conditions for the development and progression of AMD by enhancing systemic oxidative stress, impairing the stability of choroidal microvascular perfusion, and promoting inflammatory activity, which aligns with the cross-system pathological interaction proposed in the “lung–eye axis” concept. Further longitudinal cohort studies and mechanistic investigations are required to elucidate the precise role of the “lung–eye axis” in age-related macular degeneration. Collectively, the epidemiological and clinical evidence linking COPD to ocular diseases is summarized in Table 1.

**Table 1:** Key epidemiological and clinical evidence linking COPD with ocular diseases.

Ocular disease	Representative studies	Study design	Main findings	Proposed mechanisms
Dry eye disease	Stapleton et al., (10); Stern et al., (12)	Review and mechanistic studies	COPD-related inflammation may impair tear film stability and ocular surface homeostasis	Systemic inflammation, oxidative stress
Cataract	Klein et al., (19); Rim et al., (23)	Population-based studies	Higher cataract prevalence in chronic pulmonary disease populations	Oxidative stress, inflammation, corticosteroid exposure
Glaucoma	Lee et al., (36)	Cross-sectional study	Reduced lung function associated with increased open-angle glaucoma risk	Hypoxia, vascular dysregulation, RNFL injury
Diabetic retinopathy	Zhou et al., (44)	Prospective cohort study	Impaired lung function associated with higher risk of diabetic microvascular disease	Inflammation, oxidative stress, retinal ischemia
Age-related macular degeneration	Bair et al., (49)	Population-based cohort study	COPD associated with increased AMD risk	Oxidative stress, chronic hypoxia, choroidal vascular dysfunction

^*^COPD, chronic obstructive pulmonary disease; RNFL, retinal nerve fiber layer; AMD, age-related macular degeneration.

## 4 Conclusions and future directions

Integrated analysis suggests that chronic systemic inflammation, sustained or intermittent hypoxia, increased oxidative stress, and microvascular dysfunction represent the principal pathological pathways through which COPD exerts its effects on ocular tissues. In particular, the retinal microvasculature and optic nerve structures—due to their high dependence on oxygen supply and microcirculatory stability—represent the most representative ocular targets involved in the systemic effects of COPD. The application of noninvasive imaging techniques, including OCT and OCTA, has generated objective and quantitative preliminary evidence supporting this association and provides visualized support for the concept of the “lung–eye axis.”

Advances in OCT and OCTA have enabled noninvasive, high-resolution, and quantitative assessment of retinal microvascular morphology and retinal nerve fiber layer structure, providing a novel “window” into systemic microvascular and neurostructural pathology. In COPD, retinal imaging parameters—including vessel density, peripapillary capillary network morphology, and retinal nerve fiber layer thickness may serve as promising biomarkers for evaluating systemic pathological burden and alterations along the “hypoxia-vascular-neural axis,” thereby extending the “lung-eye axis” framework from mechanistic investigation toward clinical translation.

Nevertheless, current evidence remains preliminary. Most available studies are cross-sectional in design, and longitudinal data establishing causal relationships between retinal alterations and COPD progression or clinical outcomes remain limited. In addition, the specificity, sensitivity, and clinically meaningful cutoff values of these retinal imaging biomarkers have yet to be established, while technical heterogeneity across OCT/OCTA platforms further challenges reproducibility and inter-study comparability. Future efforts should therefore prioritize standardized imaging protocols and large-scale multicenter prospective cohort studies integrating pulmonary function, systemic biomarkers, and multimodal ocular imaging. Such advances may help validate the clinical utility of retinal biomarkers and facilitate their incorporation into systemic disease assessment and precision medicine strategies in COPD.

In conclusion, enhanced interdisciplinary collaboration between respiratory medicine and ophthalmology will facilitate a comprehensive understanding of the regulatory mechanisms of the “lung–eye axis,” thereby offering novel perspectives and strategies for precision stratification of COPD and the early prevention and control of its multisystem complications.
